# Complete Genome Sequence of Ferrigenium kumadai An22, a Microaerophilic Iron-Oxidizing Bacterium Isolated from a Paddy Field Soil

**DOI:** 10.1128/MRA.00346-21

**Published:** 2021-07-08

**Authors:** Takeshi Watanabe, Ashraf Khalifa, Susumu Asakawa

**Affiliations:** a Laboratory of Soil Biology and Chemistry, Graduate School of Bioagricultural Sciences, Nagoya University, Chikusa, Nagoya, Japan; b Biological Sciences Department, College of Science, King Faisal University, Al-Ahsa, Saudi Arabia; c Botany and Microbiology Department, Faculty of Science, Beni-Suef University, Beni-Suef, Egypt; University of Southern California

## Abstract

Ferrigenium kumadai An22^T^ (= JCM 30584^T^ = NBRC 112974^T^ = ATCC TSD-51^T^) is a microaerophilic iron oxidizer isolated from paddy field soil and belongs to the family *Gallionellaceae*. Here, we report the complete genome sequence of *F. kumadai* An22^T^, which was obtained from the hybrid data of Oxford Nanopore long-read and Illumina short-read sequencing.

## ANNOUNCEMENT

Microaerophilic iron-oxidizing bacteria, which are capable of oxidizing ferrous iron (Fe) under circumneutral pH and microoxic conditions, are a key player in the Fe redox cycle in environments (1). However, only a few strains of microaerophilic Fe(II) oxidizers have been identified thus far from freshwater environments (2), and their genome information has been limited. In our previous study, a novel microaerophilic Fe(II) oxidizer, Ferrigenium kumadai, isolated from paddy soil has been described (3). Here, we report the complete genome sequences of *F. kumadai* An22^T^ (= JCM 30584^T^ = NBRC 112974^T^ = ATCC TSD-51^T^).

Cultivation of strain An22^T^ and DNA preparation were described previously (3). Genome sequencing analysis was performed on the Illumina MiSeq platform (San Diego, CA) with paired-end libraries (<500 bp) and GridION with R9.4.1 flow cell (Oxford Nanopore Technologies [ONT], Oxford, UK). DNA libraries for MiSeq and ONT sequencing were prepared with a KAPA HyperPlus kit (Kapa Biosystems, Wilmington, MA) and FastGene adapter kit (Nippon Genetics, Tokyo, Japan) and with a rapid barcoding kit (ONT), respectively. Basecalling of ONT sequences was performed with Albacore v2.3.1 (ONT). Default parameters were used for all software in this study unless otherwise specified. In total, 151,075,784 (read 1, 74,493,341; read 2, 76,582,443) and 1,812,690,122 bp were obtained from MiSeq and ONT sequencing (= 58.7-fold and 704.6-fold genome coverage), respectively. The *N*_50_ value of raw ONT reads was 1,790 bp with a maximum length of 222,018 bp. Low-quality MiSeq reads (Q score, <20; single read, <127 bp) were removed with Sickle v1.33 (https://github.com/najoshi/sickle). Short ONT reads (<1,000 bp) were filtered out with Filtlong v0.2.0 (https://github.com/rrwick/Filtlong), and then error correction of the reads was carried out on FMLRC v0.1.2 (4) with the MiSeq reads as references. The quality-controlled MiSeq and ONT reads were assembled on MaSuRCA v3.2.8 (5). The assembled sequences were polished with Pilon v1.22 (6), followed by circularization of the contigs with Circlator v1.5.5 (7). The assembly quality was confirmed with BUSCO v5 (8) on gVolante v2.0.0 (9), and there was 98.63% completeness when examined with the ortholog set of *Betaproteobacteria*. Genes were annotated with DFAST v10 (10). Genes for fundamental metabolic pathways were searched on BlastKOALA v2.2 (11). The orthologous genes for Fe(II) oxidation, encoding MtoAB (12), PioAB (13), and Cyc2 (14, 15), were searched on the MBGD update 2018 (16) with the genome sequences of Sideroxydans lithotrophicus ES-1 (GenBank accession number CP001965.1), Rhodopseudomonas palustris TIE-1 (CP058907.1), Mariprofundus ferrooxydans PV-1^T^ (DS022294.1), and Acidithiobacillus ferrooxidans ATCC 23270^T^ (CP001219.1). An orthologous gene cluster table among the genome sequences used was created (17), after which candidate genes were searched with the homology search program in MBGD based on BLASTP (18).

The genome size of strain An22^T^ was 2,572,603 bp with a G+C content of 60.6%. The genome contains 2 sets of rRNA operons, 50 tRNA sequences, and 2,428 protein-coding genes, including a gene for Rubisco and a gene set for nitrogenase. A gene encoding putative Cyc2 (BBI98728.1) was found as a sole candidate gene of Fe(II) oxidation.

### Data availability.

The genome sequence of *F. kumadai* An22^T^ was deposited to the DDBJ database under the accession number AP019536.1. The raw sequencing data were deposited under BioProject accession number PRJDB7995 and SRA accession numbers DRR168795 and DRR168796.
